# A microprocessor data-capture and data-base package for the IL508 analyser

**DOI:** 10.1155/S1463924684000274

**Published:** 1984

**Authors:** Barry Clark, Brian Stewart, Robert W. Logan

**Affiliations:** 1 Department of Biochemistry Western Infirmary Glasgow G11 6NT UK; 2 Department of Biochemistry Royal Hospital for Sick Children Yorlhill Glasgow G3 8SJ UK


Journal of Automatic Chemistry, Volume 6, Number 3 (July–September 1984), pages 148–151

### A microprocessor data-capture and data-base package for the IL508 analyser

**Barry Clark**
*Department of Biochemistry, Western Infirmary, Glasgow G11 6NT, UK*

**Brian Stewart and Robert W. Logan**
*Department of Biochemistry, Royal Hospital for Sick Children, Yorkhill, Glasgow G3 8SJ, UK*

### Summary

The use of an Apple II microcomputer as an on-line data-capture device for an IL508 analyser is described. The package is equipped with a true data-base, so 650 records may be on-line at any given time for editing or printing, with a random access time of several seconds. Quality-control data is also held in the file and may conveniently be accessed for processing. The package provides for flexible output formats to the computer screen, and for printing on plain paper or sticky labels, so that each medium is optimally catered for. Facilities are also provided to send relevant data to a PDP 11/34 computer as required. This package had contributed considerably to productivity by minimizing the effort required to handle repeat analyses and samples where the date/time of collection differs significantly from the date of assay. Should the main computer be temporarily inaccessible, data may also be stored for subsequent transfer without the need for manual re-entry

### Introduction

The contribution of multichannel discrete and continuous-flow analysers to the productivity of the routine clinical chemistry department may be considerably enhanced when these instruments are equipped with suitable data-capture devices. There is little point in processing samples very quickly if there is a backlog of results requiring manual typing, either onto forms or into the main departmental computer for subsequent reporting. A previous report [1] has indicated the analytical suitability of the IL508 analyser to the needs of a paediatric biochemistry department; subsequent development work designed to integrate the analyser fully into the reporting system is described here.

The use of microcomputers provides an excellent way of solving problems associated with instrument data handling. Data-capture is 'off-line' in the traditional sense, but by equipping the system with a data-base and file-editing capability, all necessary operations such as editing, inserting repeat analyses and entering time of collection etc., can be performed on the microcomputer, and a 'clean' file of data, containing only relevant information, may then be sent to the main computer, reducing its requirement to process the data. Further, should the main computer fail, the data held on the miocrocomputer system can be used to print labels, which may be attached to forms, for issue to the wards—there is no need for manual transcription. The data is then held on disk until the main computer becomes available. Such facilities reduce demands made on reporting staff at times of stress and ensure data security.

A comprehensive package for the IL508, based on an Apple II microprocessor, is represented which incorporates the facilities discussed above. Data is captured in real-time from the analyser; calibration samples are printed but not filed. Samples and quality-control results are stored but with different codings, allowing the development of procedures for monitoring quality-control performance. Up to 650 records may be stored, and a true data-base is used to give fast, random access to any record in the file. Once data-capture is complete, records may be edited, or repeat analyses (done as 'stat' samples) inserted into the file. Selected results may then be printed as a daily log or as labels for report cards, or transmitted to the mainframe for incorporation into the laboratory records system. The microcomputer assists in this work because it is relatively simple to set up the most appropriate format for each task.

### Materials and methods

### *Computer equipment*

The package is designed to run on an Apple II microcomputer equipped with a 16K RAM language card (or an Apple //e), an 80-column card, a monitor, two disk drives and a printer. The printer interface card is the 'Grappler' (Orange Micro, 3150 E. La Palma, Suite G, Anaheim, California 92806, USA), which supports most popular dot-matrix printers. The interface to the IL508 is a CCS 7710 asynchronous serial card (California Computer Systems, 250 Caribbean Drive, Sunnyvale, California 94086, USA). The construction of the interface cable is shown in figure 1.

### *Computer software*

With the exception of the IL508 interface routine, the package is written in Apple UCSD Pascal. This language has many advantages for this type of program, not the least that it supports proper record structures and can easily be interfaced to machine-code programs. The package has two main components—data capture and data edit/print.

Correspondence should be addressed to Barry Clark.

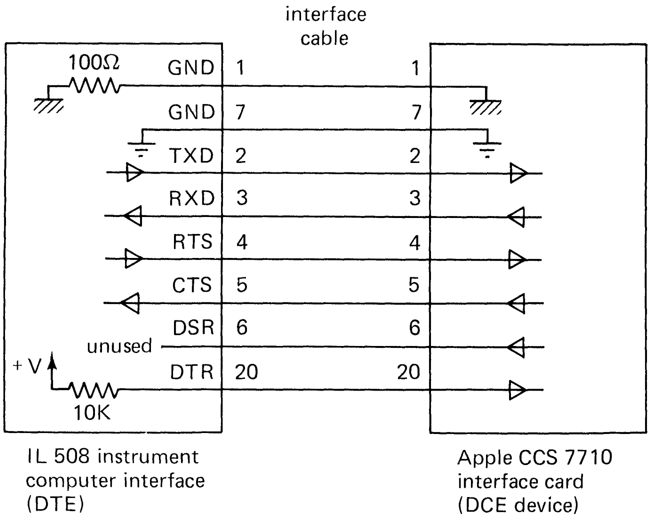

'DTE' interface pin description

| Pin # | Function |
|---|---|
| 1 | Chassis ground |
| 7 | Signal ground |
| 2 | Transmit data to Apple |
| 3 | Receive data from Apple |
| 4 | Request to send (true when IL wants to send to Apple) |
| 5 | Clear to send (acknowledgement from Apple) |
| 20 | Always true |

*Figure 1. IL508—interface cable connections to Apple microprocessor.*

(1) *Data capture*

This program reads data from the IL508 and, after suitable processing, sends information to the screen and printer and updates the data-base.

*IL508 data-capture*—this program is written in 6502 Assembler for several reasons. Data transfer from the IL508 is optimal at 1200 baud and attempting to read the CCS 7710 card from Pascal gives a maximum baud rate of 300. The default data format on the CCS 7710 is 8 data + 2 stop bits + no parity. The IL508 format is 7 data bits, 1 stop bit and even parity. It is not possible to reset the CCS card from inside a Pascal program, and this must be done using machine code. So, too, if the Apple is reading, or waiting for data from IL508, there is no simple way of interrupting this process without resetting the machine and losing the data as the Pascal 'KEYPRESS' function is disabled by the presence of the 80-column display card. The machine-code routine polls the CCS status register; if a character has been received from the IL508 then the routine stores it in a buffer. If no character is there, the Apple keyboard is polled to see if the 'Escape' key has been pressed. If it has, data capture is terminated, the buffer set to 'END' and the system files are closed down in an orderly fashion before returning to the main menu. The end of transfer of data from the IL508 is indicated by sending a ';'. Receipt of this character terminates data capture and the contents of the buffer area are made available to the Pascal program for processing.

*IL508 data processing*—after capture of a set of results from the IL508, the data must be processed. The data consists of a header string, followed by results. The format is given in table 1. The most important field is that of 'data type'. This takes the value of 0 = sample, 1 = control (QC), 2 = Auto-cal and 3 = slope cal. The latter two items are for the internal calibration of the IL508 and are shown on the screen and printer, but not held in the data-base. They also differ in that the data string has two sets of results, one pre- and one post-calibration, and so require slightly different processing. Typical screen output during a run is shown in figure 2.

*Table 1. A brief description of the data transmitted to the Apple microcomputer by the IL508 analyser. 'N' represents the position occupied by a digit. This information is captured by a machine-code routine, which passes it to the main Pascal program for processing. Much of the information does not require to be displayed and some is better expanded for display. For example, if Sample Fluid = 1 then 'urine' is displayed on the print-out; a cycle type of 1 is expanded to 'stat'.*

| Data | Brief description |
|---|---|
| : | Start of block |
| NNNN, | Computer sequence number |
| NNN, | Model ID number |
| N, | Cycle type (0 = normal or cal, 1 = 'stat') |
| N, | Units (0 = mass, 1 = SI units) |
| N, | Data type (0 = sample, 1 = control, 2 = Auto-cal, 3 = slope cal) |
| N, | Sample fluid (0 = serum or plasma, 1 = urine) |
| NN/NN/NN, | Date (month, day, year) |
| NN/NN, | Time (hours, min) |
| NN/NN/NN, | RUN/RACK/CUP numbers |
| NNNNNNNNN, | Lab ID number or QC number |
| CR LF | Carriage return and line feed |
| 'Data block 1' | Concentration values, error codes |
| CR LF | Carriage return and line feed |
| 'Data Block 2' | Calibration values and cal errors (cals only) |
| CR LF | Carriage return and line feed |
| NNN | Checksum |
| ; | End of block (complete data-set transmitted) |
| CR LF | Terminating carriage return and line feed |

IL 508 RESULTS FOR 19-SEP-83 at 09:48 HRS

| Na | K | Cl | TC02 | Urea | Creat | Calcium |
|---|---|---|---|---|---|---|
| Seq: 0353 | ID= | | 19-Sep-83 | 09:48 hrs | Autocal | – before |
| 140 | 5.0 | 98 | 25 | 17.6 | 316 | 2.65 |
| Seq: 0353 | ID= | | 19-Sep-83 | 09:48 hrs | Autocal | – after |
| 140 | 5.0 | 98 | 25 | 17.8 | 354 | 2.50 |
| Seq: 0354 | ID= | | 19-Sep-83 | 09:48 hrs | Cal/slope | – before |
| 120 | 2.0 | 76 | 15 | | | 1.87 |
| Seq: 0354 | ID= | | 19-Sep-83 | 09:48 hrs | Cal/slope | – after |
| 120 | 2.0 | 75 | 15 | | | 1.87 |
| Seq: 0357 | ID= | 01 | 19-Sep-83 | 09:53 hrs | * * URGENT | * * |
| 139 | 4.9 | 94 | 21 | 9.4 | 198 | 2.52 |
| Seq: 0358 | ID= | 31890 | 19-Sep-83 | 09:53 hrs | * * URGENT | * * |
| 132 | 3.1 | 91 | 34 | 5.2 | 33 | 2.25 |
| Seq: 0359 | ID= | 31892 | 19-Sep-83 | 09:54 hrs | * * URGENT | * * |
| 130 | 4.0 | 89 | 29 | 7.2 | 106 | 2.32 |
| Seq: 0360 | ID= | 31897 | 19-Sep-83 | 09:56 hrs | * * URGENT | * * |
| 119 | 5.4 | 84 | 15 | 34.3 | 785 | 1.95 |

*Figure 2. IL508—display of results.*

Once data processing is complete, the results and quality-control values are sent to the data-base. The data-base is based on a B + tree [2]. This is a derivative of a binary tree and ensures, given the appropriate key, rapid access to any item in the tree. The key needs to be unique for the particular record, and the IL508 sequence number (0001–9999) was chosen as a means of indexing the results. Unfortunately, circumstances may require the IL508 to be reset—system failure for example. If this occurs, it is possible to repeat the IL508 sequence numbers in the same file. To avoid problems, the data-base key is obtained by concatenating the sequence number and the laboratory number (see table 1). To allow quality-control data to be abstracted separately from patient results, the information on the 'data type' field is preserved (0 = patient, 1 = QC) in the data-base record.

All records received by the Apple are displayed on the monitor, with suitable annotations (see figure 2) to indicate the nature of the current sample (for example calibration, slope calibration, 'stat' sample). They are also transmitted to the printer to provide a log for the current period. Data capture is terminated by pressing the 'escape' key on the Apple and the user is returned to the main menu.

(2) *Data edit/print*

This program allows the user to edit data in the files either by inserting new data or by overwriting existing data. A facility for inserting date and time of sample collection (in case they differ significantly from the date and time of analysis) is provided. This is also a useful facility when performing sequential estimations on a patient, for example 'stat' electrolytes at frequent intervals. Operation of the editor is simple; the user enters the assay sequence number and the corresponding results are displayed on the screen (figure 3). Any of the displayed results may be edited, including the laboratory number. To move to the required field, the user presses the '⟨ – ' or ' – ⟩' keys. To insert a new value, the user simply starts typing; the existing value is cleared and replaced by a mask '......' (see figure 3). Pressing the 'Return' key enters the new value and the display is reformatted, with the correct number of decimal places where appropriate. Results may be deleted by entering '0'. Missing results may be inserted by moving the cursor under the appropriate heading and entering as above. Pressing the 'Escape' key at any time will terminate the edit and, if any changes have been made, the file will be updated. This saves time when entering a lot of data, there being no need to move to the end of the record to terminate the edit.

EDIT RESULTS

LN: 357264 Collected: 19-Sep-83 10.30 hrs

| Na | K | Cl | TC02 | Urea | Creat | Calcium |
|---|---|---|---|---|---|---|
| 139 | 3.... | 94 | 21 | 9.4 | 198 | 2.52 |

*Figure 3. IL508—editing of results.*

Once all editing of the file is complete, the user may then print sticky labels for inclusion in report forms. These labels are formatted to suit the existing reports and so avoid unnecessary expense in reprinting the forms. The format of the labels is shown in figure 4. The use of a dot-matrix printer allows informative text to be printed in a different font from the results, minimizing confusion when the clinician reads the report and improving the quality of information supplied to the clinician.

The user may also print a log report; its format is similar to the data-capture program. This means that an updated/edited log may be filed should subsequent verification of any report be required. When printing either labels or a log, the start and end records may be specified so that subsections of the total results file may be printed selectively.

| CHECKED BY | SODIUM mmol/l | POTASSIUM mmol/l | CHLORIDE mmol/l | $CO_2$ (Total) mmol/l | UREA mmol/l | CREATININE µmol/l | BILIRUBIN µmol/l | AST I U/l | ALT I U/l | PROTEIN (Total) g/l | CALCIUM mmol/l | PHOSPHORUS mmol/l |
|---|---|---|---|---|---|---|---|---|---|---|---|---|
| | LN 44014 | | | Collected | 29-Nov-83 | | | Assayed | 29-Nov-83 | | 14 22 hrs | |
| | 141 | 2 9 | 106 | 21 | 2.3 | 33 | | | | | 2 26 | |

*Figure 4. IL508—format of label.*

(3) *Other facilities*

From the main menu, the user may also clear existing data disks for reuse. Due to the format of the B+ tree on the disk, it is not acceptable simply to delete the file name from the disk directory—the disks must be reformatted. The Pascal format program is a separate program which returns to the Pascal operating system after use. To use it with this package and return to the menu afterwards, the menu program executes an 'EXEC' file which holds a string of commands to ensure that the program returns to the menu after formatting the data disk.

The user may also elect to transmit the data to a mainframe computer for inclusion in a reporting system. The program calls up a commercial package ('p-term professional' from Southwest Data Company Ltd, San Anee, California, USA) which ensures compatibility with most mainframes (DEC, ICL etc.) to transfer a user-selected subset of results. Full handshaking is provided so that the Apple will accommodate and accept the mainframe protocols.

### Discussion

Several factors led to the development of a sophisticated, Apple-based data-handling system for the IL508 analyser. A much simpler version had been written earlier using a WANG PCS II microcomputer. Since both data and programs were stored on tape, access to information was slow. If, during editing, an earlier result had to be edited, then the edit program needed to be terminated and the tape rewound. Such sequential access is a severe limitation to any kind of data-base. Most of these restrictions arose from the very small internal memory of around 4K RAM. The equipment was also becoming unreliable and the annual maintenance contract costs approached the price of a complete Apple II system.

Flexibility was the keynote in the redesigned system. Separate formats were required for screen display and for printing in 'log' or 'label' modes, and for sending data to the main laboratory computer. Pascal was chosen as the language most suited to this kind of application—not only is it simple to create true record structures for holding data, but the construction of complex B+ data trees for rapid data access is also catered for implicitly in the language. It is a source of concern that most laboratory information-handling programs for mini- or mainframe computers are still written in Fortran. Although this language is historically well supported, record structures can be created only by artifice and can be difficult to update to include more information. The problem is lessened by the greater speed of minicomputers, but with a microcomputer where speed is of the essence, it is sensible to choose the most appropriate language for information processing as opposed to data processing. As the current program evolved, the benefits of Pascal for rapid and simple updating became very apparent, and the power of the record structures greatly simplified modification of screen and printer outputs and the modification of record content.

It was also surprisingly simple to link the data-capture 6502 machine-code routine into the main Pascal program. This feature is well supported in Apple Pascal and workers who wish to develop such programs should consider how well the language they intend to use (Basic, Pascal, Fortran etc.) links to machine code. It is usually the case that machine code and Basic can be interfaced easily, but Basic was not used because the resulting program would have been unstructured (Applesoft Basic has no control structures such as 'WHILE' or 'CASE') and difficult to modify. Use of machine-code programs for driving interface cards to external equipment is recommended, not only for speed, but in the way that the card and the keyboard can be polled simultaneously and the appropriate action taken; stopping a program with a 'STOP' key or 'RESET' is not the most elegant way of terminating data capture. Initially, the interface card was driven by Pascal and this limited the data-transfer rate to 300 baud, as well as making it impossible to reset the card to match the format of the data arriving from the IL508. In the initial development, the only way to terminate data capture was to finish the assay with an analysis with a specified tray and cup number. This was difficult to know in advance, particularly if some analyses had to be repeated, and the system impeded the work rather than assisted it. Now the user simply presses the 'Escape' key and returns to the main menu.

The present system has been in use for around six months with relatively few problems, and the operators are pleased with the speed and ease of updating/editing existing records. The use of flexible display and printing formats has improved the clarity of information sent to the clinician. Quality-control data is stored in the same file as the results but coded differently and is, therefore, simple to extract for use in quality-control programs. A large part of a recent symposium (Clinical Computing Data Communications Group [July 1983, Uppsala]) was devoted to processing quality-control information—it was thought vital that features to handle such data are incorporated into the system and not added as after-thoughts.

Workers considering implementing similar programs should look very carefully at the language used, bearing in mind that it is often unlikely that the person who wrote the original program will be around to update it. This paper is presented to show the value of an on-line microcomputer-based data-capture data-base package as a complement to a multichannel analyser, either as a 'stand-alone' package, or interfaced to a main departmental computer. The flexibility and resilience inherent in this design are beneficial to the smooth running of the department. The paper also demonstrates the relative ease with which such information-processing programs may be written in Pascal. Being a truly structured and logical language it is much more easily updated or modified, thereby minimizing future software costs.

### References

1. Logan, R. W., Aitchison, E. M., Jamieson, E. C., Stewart, B., and Sim, F. G., *Journal of Automatic Chemistry* , **4** (1982), 7.
2. Standish, T. A., *Additional Tree Structures and their Applications in Data Structure Techniques.* (Addison-Wesley Publishing Co., 1980), 118.

### SAC 86/3rd BNASS

*20–26 July 1986, University of Bristol, UK*

The Analytical Division of the Royal Society of Chemistry has chosen Bristol as the site for the SAC 86 International Conference on Analytical Chemistry. On this occasion the SAC Conference is to be combined with the Biennial National Atomic Spectroscopy Symposium (BNASS—organized by the Atomic Spectroscopy Group of RSC/AD and the Spectroscopy Group of the Institute of Physics). SAC international conferences are held triennially and still bear the initials of the then Society for Analytical Chemistry which was responsible for their initiation.

The scientific programme will be organized around plenary, invited and contributed papers and posters covering the whole field of analytical chemistry, and all aspects of atomic spectroscopy. As at previous conferences, special symposia on particular analytical themes will be arranged by RSC groups and other associated bodies. The programme will include workshops, where research workers can demonstrate new apparatus and techniques. Update courses are also planned, to provide all-day tutorial and practical demonstration sessions. The latter will be held on the Wednesday, as an alternative to a scientific or cultural visit, or to the 3rd BNASS Programme.

*Techniques—*

Atomic spectrometry
Biochemical methods
Catalytic and kinetic methods
Chromatography
Electroanalytical methods
Electrophoresis
Enzyme techniques
Flow-injection methods
Immunoassay
Mass spectrometry
Microanalysis
Molecular spectroscopy
Photoacoustic spectrometry
Probe methods
Radiochemistry
Sample preparation
Preconcentration and separation
Thermal methods
X-ray methods.

*Materials—*

Agricultural
Atmospheric
Biological and microbiological
Clinical
Environmental
Food and drink
Geological
Industrial
Metallurgical
Particulate
Pharmaceutical
Plastics, rubbers and textiles
Surfaces
Water and effluents.

*Other aspects—*

*Automation and robotics*
Biotechnology
Chemometrics
Consumer protection
Data processing
Education
Harmonization
Historical
Microcomputers and microprocessors
Optical fibres
Quality control
Sensors
The integrated laboratory.

*The Second Circular will be ready in June 1985; to apply for it and for other information contact The Secretary, Analytical Division, Royal Society of Chemistry, Burlington House, London W1V 0BN.*

